# Impact of Care Delivered in Nondesignated Areas on Older Patients Admitted With Hip Fractures: A Quality Improvement Initiative

**DOI:** 10.1111/jep.70276

**Published:** 2025-09-21

**Authors:** Joan Solomon, Ashna Ameer, Vahida Chopda, Radcliffe Lisk, Keefai Yeong, Jay Acharya, Jonathan Robin, Christopher H. Fry, Thang S. Han

**Affiliations:** ^1^ Department of Acute Medicine Ashford and St Peter's NHS Foundation Trust Chertsey UK; ^2^ Department of Urgent and Emergency Care Ashford and St Peter's NHS Foundation Trust Chertsey UK; ^3^ Department of Orthogeriatrics Ashford and St Peter's NHS Foundation Trust Chertsey UK; ^4^ School of Physiology, Pharmacology and Neuroscience University of Bristol Bristol UK; ^5^ Department of Endocrinology Ashford and St Peter's NHS Foundation Trust Chertsey UK; ^6^ Institute of Cardiovascular Research, Royal Holloway University of London Egham UK

**Keywords:** corridor care, length of stay, mortality, ward moves, winter bed pressure

## Abstract

**Background:**

Although testimonies of devastating impacts of care delivered in nondesignated hospital areas (corridor care) are mounting, there is a paucity of quantitative data. This study aimed to assess the associations between: (1) care in nondesignated areas and key performance indicators (KPIs), including ward moves and length of stay (LOS); and (2) between KPIs and mortality.

**Methods:**

Data from this cross‐sectional study were derived from the National Hip Fracture Database audit programme (a quality‐improvement initiative commissioned by the Healthcare Quality Improvement Partnership, NHS England). In total, 508 patients (65% women) consecutively admitted with hip fractures (January 2024–January 2025) were included. The duration (h:min) of care in nondesignated areas was categorised by tertiles (< 1:20; 1:20–4:20; > 4:20). Associations between variables were determined by logistic regression, presented as odds ratios (OR) and 95% confidence intervals.

**Results:**

Patients cared for in nondesignated areas (11%) and bed care patients (89%) had similar clinical characteristics (median age = 85.5 years). Care in nondesignated areas varied inversely with seasonal average local temperatures: 10.7% in summer (22.5°C), 19.6% autumn (15.9°C), 44.6% winter (8.6°C), and 25.0% spring (14.9°C), which was more discernible than the corresponding distribution of bed care: 21.2%, 23.7%, 25.9% and 29.2%. Compared with bed care patients, those cared for in nondesignated areas for ≥ 1 h:20 min were associated with ≥ 3 ward moves: OR = 4.02 (1.61–10.06). LOS on orthogeriatric wards for bed care patients was 13.4 days, and care in nondesignated areas > 4 h:20 min was 17.2 days, which increased to 19.7 days for all patients cared for in nondesignated areas with ≥ 3 ward moves. In turn, higher in‐hospital mortality was associated with multiple ward moves: OR = 2.63 (1.23–5.66) and prolonged LOS: OR = 3.23 (1.53–6.81).

**Conclusions:**

The impact of care delivered in nondesignated areas exposed by KPIs is consistent with testimonies from patients and NHS staff. This evidence serves as a stimulus to take urgent action to abolish care in nondesignated areas.

## 1 Introduction

The delivery of care for patients in nondesignated areas (also referred to as corridor care) includes locations such as corridors, car parks, and even converted storerooms and offices at its most desperate. This has become common practice in the National Health Service (NHS), which is escalated when the occupancy rates are high and there is inadequate bed space [1, 2]. Care in nondesignated areas is unsafe since critical facilities for monitoring equipment and treatment are not accessible while infection control is compromised. Furthermore, confidential medical assessment and treatment, as well as basic needs including feeding, hygiene and toileting, are conducted in public [1, 2]. Many such patients are vulnerable older adults who have multiple underlying chronic illnesses while waiting in pain and disorientation [3]. This issue has been raised repeatedly by patients [4] and healthcare professionals [1, 2, 5]. Care for patients in nondesignated areas has become a systematic widespread crisis in the NHS and, worryingly, accepted as a national ‘norm’. A recent survey of NHS doctors conducted by the Royal College of Physicians revealed 78% of respondents had provided care for patients in a temporary environment in the past month [1]. Consequently, this practice has a serious impact on the well‐being of healthcare professionals who feel demoralised, stressed and powerless.

Calls to end the practice of care for patients in nondesignated areas have been raised repeatedly by patients [4, 6] and healthcare professionals [1, 2, 5]. However, little could be achieved because information on this practice has not been formally documented, with consequential lack of insight into the aetiology of this problem. This triggered the Royal College of Physicians to issue a recent statement calling on the NHS and UK government to: ‘formally measure and nationally report on how many patients are being treated in temporary care environments all year round; put systems and processes in place to eliminate such corridor care; and support patients and staff when care is delivered in temporary care environments’ [7]. In this study of older patients admitted with hip fractures, the primary objective was to examine the impact of care for patients in nondesignated areas on the quality of patient care using two key performance indicators (KPIs), ward moves and hospital length of stay (LOS). A secondary objective was to relate these two KPIs to in‐hospital mortality in this cohort of patients.

## 2 Methods

### 2.1 Study Design, Participants and Setting

This cross‐sectional study recruited individuals consecutively admitted to an NHS hospital with hip fractures (January 2024–January 2025). This study participates in the National Hip Fracture Database (NHFD) audit programme, which is a quality‐improvement initiative commissioned by the Healthcare Quality Improvement Partnership, which in turn is commissioned by NHS England. This audit programme is managed by the Royal College of Physicians, and data are collected under section 251 of the NHS Act 2016 following approval by the Health Research Authority Confidentiality Advisory Group (CAG 8‐03(PR11)/2013) [8].

### 2.2 Data Collection

Data were prospectively collected by a Trauma Coordinator, comprising demographic factors including age and sex, and nutritional status using the Malnutrition Universal Screening Tool (MUST) protocol [9]. Patients were classified into three groups: well‐nourished (patients without evidence of malnutrition or malnourishment); high risk of malnutrition for patients with a MUST score ≥ 2; or malnourishment for those who met the NICE criteria [10]. Prefracture mobility status was assessed by a standardised tool [11, 12]. The duration of care in nondesignated areas was categorised into tertiles. The quality of patient care was recorded for two KPIs: ward moves and LOS spent on an orthogeriatric ward, as well as in‐hospital mortality. Climate records were obtained from a local meteorological station (Wisley, Surrey), which provided monthly average temperatures over the most recent three decades (1991–2020) [13]. Seasons were based on British meteorological definitions: spring (March to May), summer (June to August), autumn (September to November), and winter (December to February). Types of hip fracture were defined according to the Garden classification and grouped into intracapsular fractures, extracapsular fractures, and peri‐prosthetic failure [14].

### 2.3 4AT Assessment

The 4AT was measured within 1 day after hip surgery, including: ‘Alertness’, ‘AMT4’, ‘Attention’, and ‘Acute change or a fluctuating course’ [15]. The scores obtained from the components of the 4AT were summated to produce a composite score (0: unlikely to have delirium or severe cognitive impairment; 1–3: possible cognitive impairment, which does not exclude the possibility of delirium; ≥ 4: possible delirium ± cognitive impairment) [15].

### 2.4 Statistical Analysis

Differences between categorical variables were tested by *χ* ^2^ tests and for continuous variables by analysis of variance. Data sets that demonstrated a skewed distribution were logarithmically transformed. Multivariable logistic regression was used to determine the risk of multiple ward moves (dependent variable) for those spending a longer duration in nondesignated areas (independent variable), with reference to those in continuous bed care. Results are expressed as odds ratios (OR) and 95% confidence intervals (CI) and presented in two models: unadjusted and adjusted for potential confounding factors including age, sex, 4AT scores, nutritional status, prefracture mobility, types of fracture and seasonality. The statistical significance threshold was accepted as *p* < 0.05. All statistical analyses were performed using SPSS® Statistics for Windows, Version 28.0 (IBM Corp).

## 3 Results

### 3.1 Patient Characteristics

In total, 508 patients (67% women) of median age of 85.5 years (interquartile range: 79.5–90.9) were analysed. There were 89% of patients receiving bed care throughout and 11% in nondesignated areas. Tertiles of duration of care in nondesignated areas were < 1 h:20 min, 1 h:20 min–4 h:20 min, and > 4 h:20 min (minimum = 1 min, maximum = 21.7 h), and the median LOS on an orthogeriatric ward was 13.5 days (interquartile range: 8.4–20.1). Table 1 shows the clinical characteristics of patients: 17.5% had 4AT scores 1–3, and 17.3% 4AT scores ≥ 4. There were 80.3% of patients considered as well‐nourished, 14.4% at risk of malnutrition, and 4.9% at risk of malnourishment. The distribution of seasonal admissions was lowest in spring (21.7%), rising progressively to 22.2% in summer, 25.2% in autumn and peaked at 30.9% in winter. Intracapsular fractures (53.9%) comprised the largest proportion of all hip fractures, followed by extracapsular fractures (36.2%) and peri‐prosthetic failures (9.8%). A total of 95.3% underwent hip operation and 5.9% died in hospital. Age, sex distribution, 4AT scores, nutritional status, prefracture mobility and types of hip fracture did not differ between care in nondesignated areas and bed care groups (Table 2).

**Table 1:** Characteristics of 508 patients admitted with hip fractures between January 2024 and January 2025.

	*n*	%
Sex distribution		
Women	339	66.7
Men	169	33.3
Bed status		
Bed care	452	89.0
Care in nondesignated areas	56	11.0
4AT scores		
0	262	51.6
1–3	89	17.5
≥ 4	88	17.3
Unknown	69	13.6
Nutritional status		
Well‐nourished	408	80.3
At risk of malnutrition	73	14.4
Malnourished	25	4.9
Unknown	2	0.4
Prefracture mobility		
Freely mobile without aids	177	34.8
Mobile outdoors with one aid	119	23.4
Mobile outdoors with two aids or frame	114	22.4
Some indoor mobility but never goes outside without help	79	15.6
No functional mobility	9	1.8
Unknown	10	2.0
Seasons		
Spring	110	21.7
Summer	113	22.2
Autumn	128	25.2
Winter	157	30.9
Fracture type		
Intracapsular fractures	274	53.9
Extracapsular fractures	184	36.2
Peri‐prosthetic failures	50	9.8
Hip operation	484	95.3
Death	30	5.9

*Note:* 4AT: Alertness, AMT4, Attention, and Acute change or a fluctuating course.

**Table 2:** Characteristics and outcomes among patients receiving care in nondesignated areas and those receiving continuous bed care.

	Time spent in nondesignated areas (h:min)				
	0:00 (bed care)	< 1:20	1:20–4:20	> 4:20	
	Median (IQR)	Median (IQR)	Median (IQR)	Median (IQR)	Kruskal–Wallis test
Age (years)	85.5 (79.5–90.9)	85.4 (82.2–90.0)	84.0 (78.4–94.1)	87.9 (79.9–91.2)	0.482

	**%**	**%**	**%**	**%**	*χ* ^2^ test
Sex distribution					
Women	67.3	55.6	73.7	57.9	0.542
Men	32.7	44.4	26.3	42.1	
4AT scores					
0	50.7	55.6	63.2	57.9	0.897
1–3	18.1	11.1	15.8	10.5	
≥ 4	17.7	11.1	10.5	21.1	
Unknown	13.5	22.2	10.5	10.5	
Nutritional status					
Well‐nourished	80.3	83.3	78.9	78.9	0.948
Risk of malnutrition	14.4	11.1	10.5	21.1	
Risk of malnourishment	4.9	5.6	10.5	0	
Unknown	0.4	0	0	0	
Prefracture mobility					
Mobility outdoor	81.7	88.2	89.5	84.2	0.745
Mobility limited to indoor	18.3	11.8	10.5	15.8	
Fracture type					
Intracapsular fractures	52.9	61.1	68.4	57.9	0.722
Extracapsular fractures	36.9	27.8	31.6	31.6	
Peri‐prosthetic failures	10.2	11.1	0	10.5	

### 3.2 Seasonal Effects

**Figure 1:**
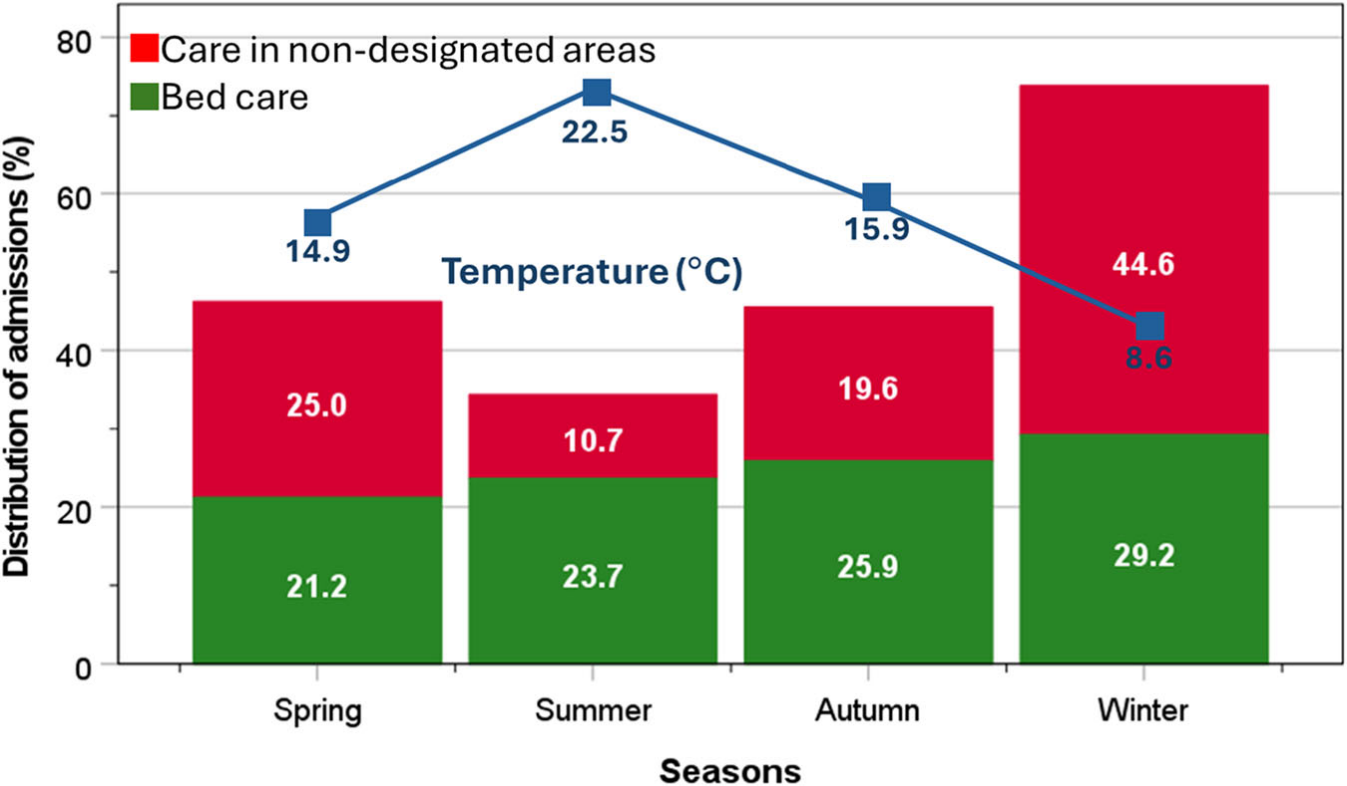
Seasonal variation in admissions among patients receiving bed care throughout and those receiving care in nondesignated areas.

The distribution of care for patients in nondesignated areas was related to average local temperatures over the four seasons: 10.7% in summer (22.5°C), 19.6% autumn (15.9°C), 44.6% winter (8.6°C), and 25.0% spring (14.9°C), which was more discernible than the corresponding seasonal distribution of total bed care: 21.2%, 23.7%, 25.9% and 29.2% (Figure 1).

### 3.3 Primary Outcomes

**Figure 2:**
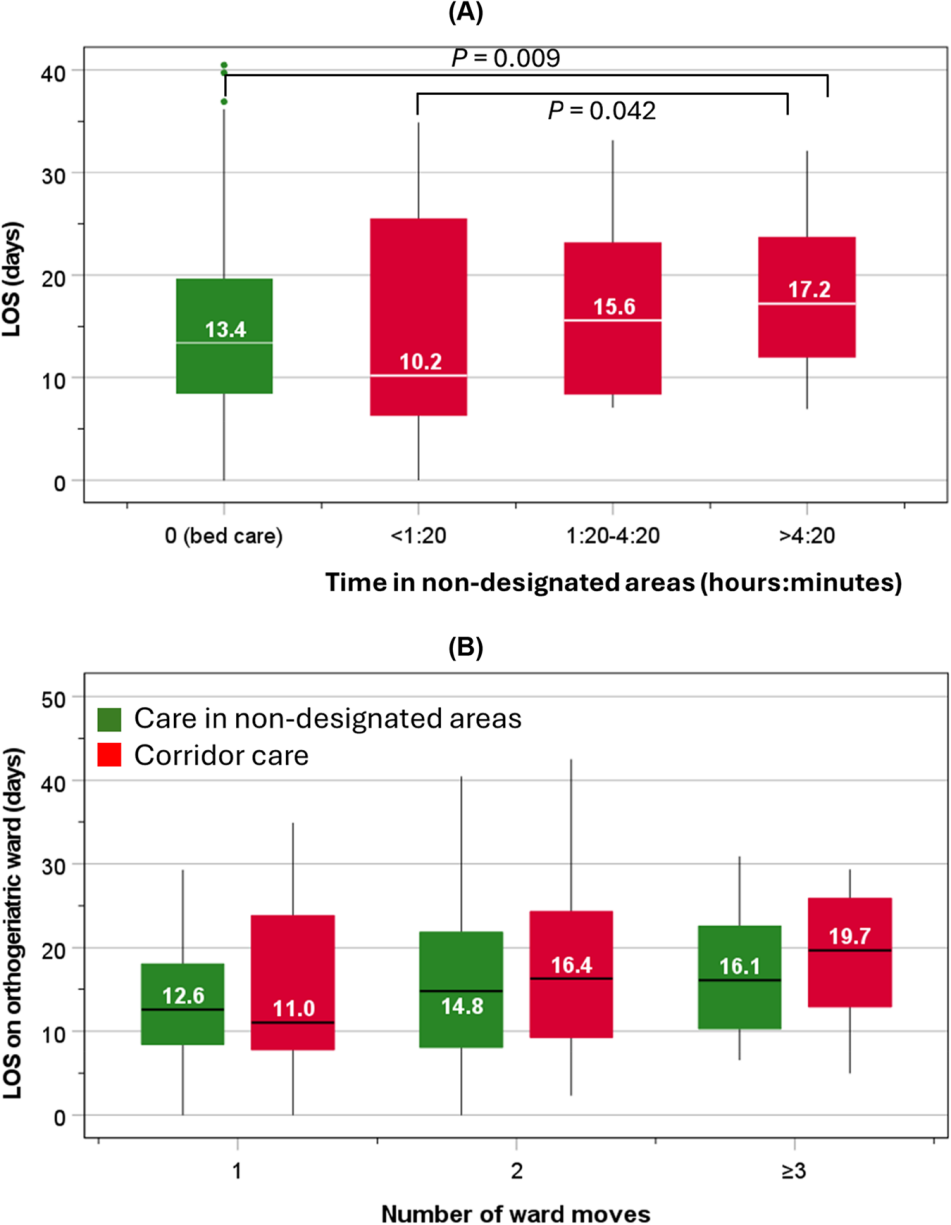
Length of stay among patients receiving bed care and those receiving care in nondesignated areas at different durations (A) and the number of ward moves (B).

Compared with bed care patients, those with care in nondesignated areas for ≥ 1 h 20 min were associated with having ≥ 3 ward moves: rates = 18.4% versus 5.3%; OR = 4.02 (1.61–10.06, *p* = 0.003). The median LOS for bed care patients was 13.4 days, and for those with care in nondesignated areas increased progressively from 10.2 to 15.6 days, and to 17.2 days for patients spending in nondesignated areas < 1 h 20 min, 1 h 20 min–4 h 20 min, or > 4 h 20 min, respectively. This was further extended to 19.7 days among all patients in nondesignated areas who had ≥ 3 ward moves (Figure 2). The rates of in‐hospital mortality did not differ (*p* = 0.426) between bed care patients (5.8%) and all patients who had care delivered in nondesignated areas (7.1%).

### 3.4 Secondary Outcomes

**Figure 3:**
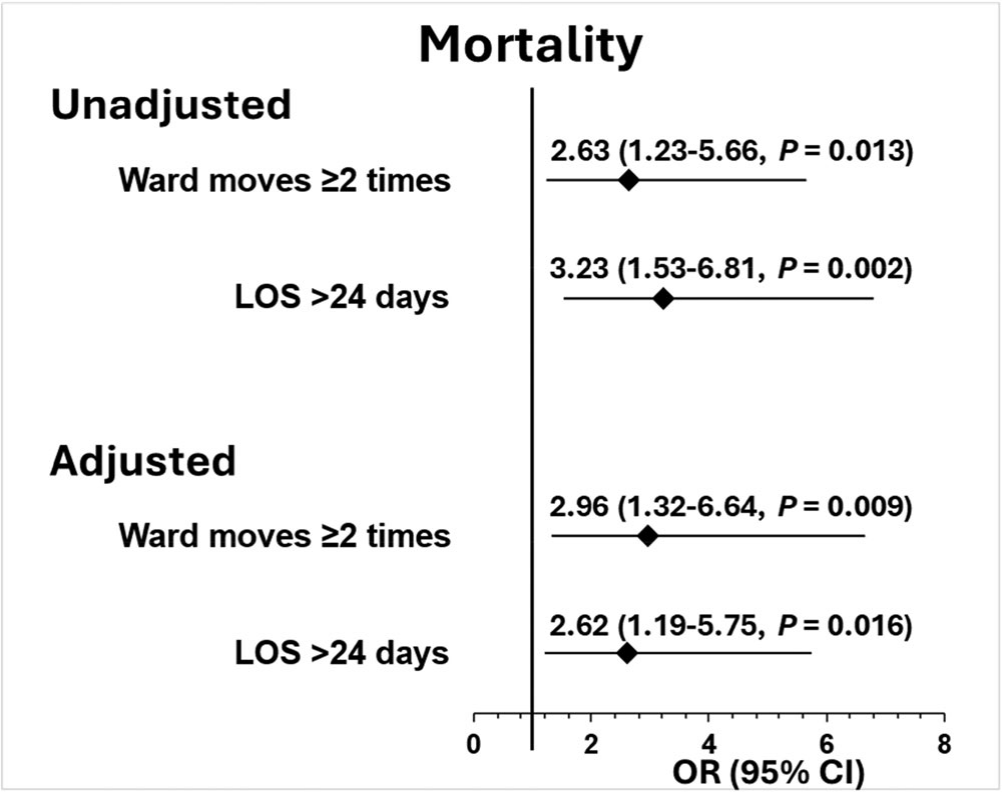
Association of in‐hospital mortality with multiple ward moves and prolonged LOS among 508 patients admitted with hip fractures, presented as unadjusted and adjusted for age, sex, 4AT scores, nutritional status, prefracture mobility, types of fracture and seasonality.

Among all patients admitted with hip fractures, higher in‐hospital mortality was associated with those with ≥ 2 ward moves: OR = 2.63 (95% CI = 1.23–5.66) compared with those with one ward move. Staying on an orthogeriatric ward > 24 days was also associated with higher mortality: OR = 3.23 (95% CI = 1.53–6.81) compared with those staying < 24 days. Additional adjustment for age, sex, 4AT scores, nutritional status, prefracture mobility, types of fracture and seasonality changed the ORs only slightly (Figure 3).

## 4 Discussion

### 4.1 Key Findings

In this study of older patients admitted with hip fractures, we observe that patients who received care in nondesignated areas were more common in colder seasons. Patients cared for in nondesignated areas and bed care patients had similar clinical characteristics. The primary objective results revealed that a longer duration of care in nondesignated areas was associated with high numbers of ward moves and longer LOS on an orthogeriatric ward. In addition, high numbers of ward moves extended LOS by an extra 2.5 days among patients cared for in nondesignated areas. To the best of our knowledge, this study of an association between care for patients in nondesignated areas and the two KPIs (ward moves and LOS) has not been reported in the literature. The secondary objective showed an association of higher mortality by 2.6‐fold in those who had ≥ 2 bed moves and 3.2‐fold in those who stayed > 24 days on the geriatric ward.

### 4.2 Characteristics of Patients Cared in Nondesignated Areas

Age, sex distribution, 4AT, nutritional status and preadmission frailty index, and types of fracture or seasonality did not differ between bed care and care in nondesignated areas. This evidence indicates the absence of bias for allocating patients to temporary escalation areas—therefore differences in KPI (see below) are mainly attributable to care itself in nondesignated areas, rather than their clinical status. However, it should be borne in mind that care in nondesignated areas is not suitable for any patient, especially for patients with hip fractures. These patients are highly vulnerable due to their advancing age and increased frailty with many underlying chronic conditions. Such patients suffer excruciating pain and with a high risk of delirium at admission [16].

### 4.3 Seasonal Effects on Care in Nondesignated Areas

The annual cycle of winter crisis showed no sign of abating [17]. The present study found care for patients in nondesignated areas occurs throughout the year but is significantly exacerbated in colder seasons. This observation provides an indirect evidence of NHS system strain during predictable seasonal surges, which reflects the larger scale of crisis encountered by the NHS. Colder months have always been challenging and a huge burden on healthcare services. This is mostly due to a greater number of older adults admitted with winter‐related conditions such as respiratory, gastrointestinal and urinary tract infections, especially those with multiple underlying chronic conditions [18]. High admissions during colder months are compounded by delays in hospital discharge of patients back to their own home. Winter infections also affect healthcare workers leading to staff shortages. To mitigate problems encountered by NHS staff during these challenging periods, several approaches have been recommended, including transparency in raising concerns and exposing risks, learning and improvement, and a supportive culture in a high‐pressure environment [4].

### 4.4 Care in Nondesignated Areas and KPIs

The present study focussed on ward moves and LOS as KPIs in relation to care in nondesignated areas. These two KPIs are important as they are commonly used in the NHS to measure efficiency and quality of patient care [19, 20]. It should be noted that ward moves and LOS are inextricably linked to one another; that is, high numbers of ward moves increase the risk of LOS, and possibly vice versa. The observation of multiple ward moves and longer LOS among those receiving care in nondesignated areas is relevant, highlighting the potential risk associated with these two KPIs.

Indeed, our finding of a higher mortality associated with multiple ward moves among all patients admitted with hip fractures agree with findings of health complications observed in other studies. One study of 566 patients admitted to a tertiary hospital found that those who were moved ≥ 3 times had an increased risk of a recorded ‘adverse event’ (not specified), compared with those moved fewer times. The same study found that the levels of satisfaction, anxiety or delirium were not related to the frequency of ward moves [21].

Several studies found an increased number of ward moves was associated with a greater risk of falls [22, 23], and wound infections among surgical patients [22]. It is thought that poor communication between discharging and admitting wards, and staff time pressures around ward moves were considered as potential factors for an increased risk of falls and the level of stress with patients [23]. Ward moves out of working hours is also harmful. A study of patients discharged from an intensive care unit out‐of‐hours were commonly discharged prematurely, had inadequate handover, were physiologically unstable, and deterioration was not recognised or escalated appropriately [24].

### 4.5 Association of KPIs With Mortality and LOS

The underlying reasons for an excess mortality associated with multiple ward moves are unclear, but it is possible that patients who received care in nondesignated areas may be overlooked or neglected as communication is difficult. Care in nondesignated areas may increase the risk of medication errors or delay in administering time critical medications (MISSED): Movement disorders (Parkinson's/Myasthenia); Immunomodulators (HIV medication); Sugar (diabetes medications such as insulin); Steroids (adrenal insufficiency); Epilepsy (anticonvulsants); and Direct oral anticoagulants and warfarin [25]. Multiple ward moves are more time‐consuming as they require multiple hand‐overs. Frequent ward moves also diminish continuation of patient care. This may explain the reason for an association between multiple ward moves and the longer LOS observed in this study, and with those from previous studies [26]. Nonclinical patient moves can be harmful. They predominantly affect older, frailer patients, and increase the risk of falls, delirium, medication errors and extend LOS [26].

Findings of prolonged LOS and mortality observed in this study are also in line with those from previous studies of patients admitted to hospital for general medical conditions [27] and for hip fractures [28]. A longer LOS is also associated with an increased risk of nosocomial infections and early readmission [27]. In addition, hospitalisation leads to weakness—each day in hospital reduces muscle strength by 5% [29]. Furthermore, prolonged LOS increases the cost to healthcare services. The cost of stay in hospital is about £300 per person a day [30].

We included types of hip fracture as a potential confounding factor in the adjusted logistic model since the types of hip fracture associate differently with outcomes—extracapsular fractures are associated with greater mortality and prolonged LOS in hospital, possibly due to a more severe injury or suboptimal management of this type of fracture [31]. In a recent study from our group, we observed that compared with women with intracapsular fractures, women with extracapsular fractures were associated with greater risks of malnutrition and malnourishment, stroke, and diabetes, as well as being less likely to be discharged to their own homes and more likely to be readmitted to hospital within 30 days of discharge [14]. However, the present study found no significant differences between types of hip fracture and the duration of care in nondesignated areas, possibly due to the small sample. Further studies of larger samples are therefore required to determine outcomes in such groups of patients whose care are delivered in nondesignated areas.

### 4.6 Intervention

A recent review by Cantrell et al. found few initiatives specifically identified as a response to winter pressures [17]. Although discharge to assess (D2A) and hospital‐at‐home interventions were well‐supported, there was limited evidence for other responses. There was also a lack of studies considering patient, family and provider needs when developing interventions aimed at improving delayed discharge, or measuring the longer‐term impact of interventions. Within our hospital, we have recently established a ‘South‐East Region (England) Frailty Community Practice’ for geriatric care. This service is by a consultant geriatrician who has brought clinicians together to share good practice, follow current national guidelines and secure support from the regional team. This initiative has enabled our Frailty team to embrace the FRAIL strategy [32], which outlines five key principles of an acute frailty service, including: *Focussing* on the acute problem with the aim of strengthening Same‐Day Emergency Care (SDEC) as a default approach. This ensures clarity on who can *Refer*; aiming to undertake a comprehensive geriatric *Assessment*; *Identifying* needs early for those with Clinical Frailty Scale scores ≥ 7 who would benefit from advanced care planning; and ensuring our patients *Leave* in a timely fashion with discharge summaries within 24 h. The effectiveness of this service is being audited to assess for quality care improvement, including a reduction in care for patients in nondesignated areas. Implementation of an in‐hospital orthogeriatric service has been shown to play a crucial role in improving outcomes of patients admitted with hip fractures. These include health‐related quality of life [33], reducing LOS and mortality in hospital among the oldest group (> 90 years), as well as a reduction in new discharge to nursing care [34].

## 5 Limitations

We selected older patients admitted with hip fractures in this study to emphasise that even one of the most vulnerable groups of patients was at risk of being allocated to temporary escalation areas. Caution should be taken when interpreting the findings from this study, and the results should not be extrapolated to general populations. However, based on evidence from our study, it would be prudent to minimise the practice of care in nondesignated areas for all patients attending the Accident and Emergency department. The mortality rate was relatively low for the given sample in this study; larger samples and longer duration of follow‐up are necessary in future studies to confirm these findings, or otherwise.

In conclusion, the impact of care for patients in nondesignated areas, exposed by KPIs as studied here, sheds light on the testimonies reported by patients, relatives and NHS staff. This evidence serves as a stimulus for governments to take urgent action to abolish care delivered in nondesignated areas—to preserve patient safety and dignity, as well as the wellbeing of healthcare professionals.

## Ethics Statement

The authors have nothing to report.

## Conflicts of Interest

The authors declare no conflicts of interest.

## Data Availability

The authors have nothing to report.
